# Gender Difference in the Interaction Effects of Diabetes and Hypertension on Stroke among the Elderly in the Shih-Pai Study, Taiwan

**DOI:** 10.1371/journal.pone.0136634

**Published:** 2015-08-31

**Authors:** Yun-Ju Lai, Hsi-Chung Chen, Pesus Chou

**Affiliations:** 1 Division of Endocrinology and Metabolism, Department of Internal Medicine, Puli Branch of Taichung Veterans General Hospital, Nantou, Taiwan; 2 Institute of Public Health and Community Medicine Research Center, National Yang-Ming University, Taipei, Taiwan; 3 School of Medicine, National Yang-Ming University, Taipei, Taiwan; 4 Department of Psychiatry and Center of Sleep Disorders, National Taiwan University Hospital, Taipei, Taiwan; The Ohio State University, UNITED STATES

## Abstract

**Aims:**

To investigate the interaction effects of diabetes and hypertension on stroke, and also investigate the independent and interaction effects of parental history and environmental factors on diabetes and hypertension in a cross-sectional elderly population.

**Methods:**

The Shih-Pai Community Medical Service Program was a community-based, fixed cohort study conducted between June 1999 and November 2002. Socio-demographic and clinical data of subjects aged 65 years and older were collected by well-trained interviewers during home visits. Interaction effects were analyzed using Rothman’s synergy index (SI).

**Results:**

In total, 4,124 subjects were included in the study, with 2,284 males and 1,840 females. The synergistic interaction of diabetes and hypertension on stroke was statistically significant in women (SI = 3.16, 95% CI: 1.35–7.39). The synergistic interaction of parental diabetes and being overweight on diabetes was only statistically significant in men, and not in women (SI = 3.30, 95% CI: 1.00–10.83 in men, and SI = 1.15, 95% CI: 0.30–4.39 in women).

**Conclusions:**

A synergistic interaction was found for diabetes and hypertension in both sexes when parental history and being overweight were combined. Furthermore, combining diabetes and hypertension in elderly women was significant in terms of the risk of stroke. Strategies to control risk factors in individuals at additional high risk are urgently needed.

## Introduction

In almost every country worldwide, the number of people aged over 65 years is rapidly growing. The prevalence of stroke has also risen rapidly in the elderly, not only significantly threatening their health, but also leading to huge amounts of health care spending. Diabetes and hypertension are major risks of stroke [1]. Interventions that combat the risk of diabetes and hypertension are important to prevent stroke. Genetic and environmental factors combine to contribute to the development of diabetes and hypertension. Many studies have investigated independent effects of genetic and environmental factors on diabetes and hypertension, but interaction effects between genetic and environmental factors on stroke are highly important and not well investigated [2].

Previous studies have shown that a parental history of diabetes better reflects the genetic phenotype than a general family history [3]. Families tend to have similar lifestyles, including dietary and exercise habits that affect the development of type 2 diabetes. It is therefore difficult to establish whether diabetes results from similar lifestyle factors or a genetic predisposition. Furthermore, factors such as family size and the age and lifestyle of siblings may bias the interpretation of genetic effects [4]. Thus, parental history can reflect genetic influence more accurately than family history.

Environmental factors contribute to the onset of type 2 diabetes mellitus and hypertension, including lifestyle factors, such as being overweight, physical inactivity, smoking, and dietary habits [3–7]. These environmental risks are preventable and modifiable. In addition to their independent contributions, the interaction effects of genetic and environmental factors should be considered [8–13], but have not been fully investigated. Furthermore, reported interaction effects between parental medical history and environmental factors are diverse in their conclusions and such studies are sparse.

Rothman et al. reported the distinction between biological interactions and statistical outcomes [14]. The resulting synergy index (SI) and relative excess risk as a result of such interactions can be used to test whether the joint effects of two distinct risk factors is greater than the sum of the independent effects of single factors on disease development. The analysis of interactions between parental and environmental factors can identify important environmental factors that are particularly relevant to the development of diabetes or hypertension. In this study, we aimed to assess the interaction effects of diabetes and hypertension on stroke, and also investigate the independent and interaction effect of parental history and environment factors on diabetes and hypertension using baseline data from the Shih-Pai study.

## Materials and Methods

The institutional review board of the Taipei Veterans General Hospital approved this study and the consent procedure. Participants had provided their written informed consent to participate in this study. All authors declare that they have no conflict of interest.

### Study site and participants

The Community Medicine Research Center of the National Yang-Ming University conducted the Shih-Pai Community Medical Service Program, which was a community-based survey. Subjects were recruited between June 1999 and November 2002 in the Shih-Pai area of Taipei, Taiwan. Of the total of 9,175 residents aged 65 years and older, 1,284 people had changed address, 175 had died, and 557 were either institutionalized or too disabled to participate. In total, 7,159 subjects were interviewed and 4,124 subjects were analyzed after cases with missing data were excluded.

### General assessment

Demographic data, such as age, sex, height, body weight, educational level, marital status, and living arrangements, were collected by well-trained interviewers during home visits. Approximately 40% data of body height and weight were missing because the elderly individuals were too weak to stand or sit on the weighing machine. Information regarding smoking habits (current, former, or never smokers) and alcohol consumption (current, former, or never use) were collected through direct questioning during the face-to-face interview. Former and never smokers were classified as non-smokers and former and never alcohol consumption was classified as no alcohol consumption due to the small sample sizes.

### Definition of chronic disease and parental history

A self-reported medical history with respect to diabetes, hypertension, cardiovascular disease, and stroke were collected. Parental history as defined by self-reported answers were “yes” to “I have the disease”, and “no” to “I do not have the disease”. Personal history was defined only if both self-reported answers were “yes” to “I have the disease” and “I have received related treatment”.

### Statistical analysis

Statistical analyses were performed using the SAS version 9.3 statistical software. Pearson’s χ^2^ test was used to examine significant associations between categorical variables. Two sample t-tests were used to examine significant associations between continuous variables. Multiple logistic regression analysis was performed to identify risks in diabetes, hypertension, and stroke. Important measures such as age, diabetes, hypertension, being overweight, and parental history were chosen for adjustments. Rothman’s synergy index was used to investigate the biological interactions between parental diabetes and hypertension, parental hypertension and overweight, and diabetes and hypertension. An SI score >1.0 indicated a significant synergistic interaction and an SI score <1.0 indicated antagonism [15, 16]. The confidence interval (CI) of synergy index was estimated with the method. All *p*-values were two-sided and the level of significance was set at 0.05.

## Results

The characteristics of the study populations are presented in Table 1. In total, 4,124 subjects were analyzed, of which 2,284 were male and 1,840 were female. Among the study population, men were significantly older (74.2 ± 5.8 years vs. 73.6 ± 5.8 years, p = 0.001). The current definition proposed by the Taiwan Health Promotion Administration, Ministry of Health and Welfare designates Taiwanese with a body mass index (BMI) of 24 or more as overweight. Men tended to smoke (27.3% vs. 4.3%, p < 0.001) and drink alcohol more commonly than women (5.2% vs. 1.6%, p < 0.001). The prevalence of parental diabetes (4.7% vs. 6.6%, p = 0.02), parental hypertension (12.6% vs. 16.5%, p = 0.02), and diabetes (15.8% vs. 13.5%, p = 0.04) were higher in women than in men. The prevalence of stroke (4.7% vs. 3.0%, p = 0.01) was higher in men than in women.

**Table 1:** Clinical characteristics of participants (n = 4124).

	Total	Male (n = 2284)	Female (n = 1840)	*P*
Age(years)	74.0±5.8	74.2±5.8	73.6±5.8	0.001
65–69	1231(30.3%)	633(27.9%)	598(33.3%)	
70–74	1300(32.0%)	765(33.7%)	535(29.8%)	
≥75	1534(37.7%)	870(38.4%)	664(37.0%)	
Body mass index≥24(kg/*m* ^2^)	1368(55.7%)	827(55.6%)	541(55.8%)	0.90
Smoking	697(17.2%)	620(27.3%)	77(4.3%)	<0.001
Alcohol	373(9.2%)	345(15.2%)	28(1.56%)	<0.001
Parental diabetes	177(5.6%)	80(4.7%)	97(6.6%)	0.02
Parental hypertension	455(14.4%)	214(12.6%)	241(16.5%)	0.002
Diabetes	581(14.5%)	301(13.5%)	280(15.8%)	0.04
Hypertension	1688(41.8%)	930(41.2%)	758(42.5%)	0.38
Stroke	160(4.0%)	106(4.7%)	54(3.0%)	0.01

Diabetes (OR = 2.01, 95% CI: 0.99–4.07 in men) and hypertension (OR = 3.18, 95% CI: 1.65–6.14 in men; OR = 6.73, 95% CI: 2.14–21.15 in women) are significant risks for stroke (Table 2). For men, a positive parental history and being overweight (BMI ≥24) were independently associated with an increased odds ratio for diabetes. Men who were overweight (OR = 2.48, 95% CI: 1.60–3.85), hypertensive (OR = 2.77, 95% CI: 1.84–4.16), or had a parental history of diabetes (OR = 8.19, 95% CI: 4.24–15.84) had a significantly increased risk for diabetes. Both men and women who were overweight (OR = 1.91, 95% CI: 1.46–2.51 in men; OR = 2.06, 95% CI: 1.49–2.85 in women), had diabetes (OR = 2.76, 95% CI: 1.84–4.10 in men, and OR = 2.91, 95% CI: 1.84–4.61 in women), or had a parental history of hypertension (OR = 4.96, 95% CI: 3.26–7.54 in men; OR = 4.96, 95% CI: 3.26–7.56 in women) had a significantly higher risk of hypertension.

**Table 2:** Multiple logistic regressions for factors associated with stroke, diabetes, and hypertension.

	Stroke				Diabetes				Hypertension			
	Male		Female		Male		Female		Male		Female	
	OR	(95% CI)	OR	(95% CI)	OR	(95% CI)	OR	(95% CI)	OR	(95% CI)	OR	(95% CI)
Age(years)												
70–74 v.s. 65–69	0.86	(0.41–1.80)	0.72	(0.25–2.11)	1.06	(0.66–1.70)	1.23	(0.74–2.06)	1.34	(0.96–1.87)	1.29	(0.88–1.90)
≥75 v.s. 65–69	1.10	(0.54–2.22)	0.93	(0.33–2.65)	1.07	(0.66–1.72)	0.91	(0.52–1.58)	1.21	(0.87–1.69)	1.29	(0.87–1.89)
Body mass index≥24(kg/*m* ^2^)	1.11	(0.59–2.08)	1.20	(0.47–3.07)	2.48	(1.60–3.85)	1.28	(0.81–2.01)	1.91	(1.46–2.51)	2.06	(1.49–2.85)
Smoking(yes v.s. no)	1.04	(0.53–2.02)	-	-	1.10	(0.71–1.71)	1.74	(0.65–4.65)	0.96	(0.70–1.31)	0.38	(0.15–0.96)
Alcohol(yes v.s. no)	1.70	(0.85–3.38)	-	-	1.00	(0.60–1.67)	1.32	(0.25–7.08)	1.27	(0.88–1.82)	1.38	(0.34–5.61)
Parental diabetes(yes v.s. no)	0.59	(0.13–2.73)	1.56	(0.39–6.28)	8.19	(4.24–15.84)	5.15		0.32	(0.15–0.67)	0.73	(0.37–1.41)
Parental hypertension(yes v.s. no)	1.31	(0.62–2.78)	0.83	(0.30–2.34)	1.09	(0.65–1.81)	0.61	(0.34–1.08)	4.96	(3.26–7.54)	4.96	(3.26–7.56)
Diabetes(yes v.s. no)	2.01	(0.99–4.07)	1.99	(0.73–5.42)					2.76	(1.84–4.10)	2.91	(1.84–4.61)
Hypertension(yes v.s. no)	3.18	(1.65–6.14)	6.73	(2.14–21.15)	2.77	(1.84–4.16)	2.86	(1.82–4.51)				

Odds ratio cannot be computed because in the stroke group, the number of female participants with smoking habits and alcohol consumption were too few.

When diabetes was combined with hypertension in elderly, the risk of stroke increased much more in women than in men (OR = 3.91, 95% CI: 2.08–7.35 in men; OR = 17.42, 95% CI: 7.18–42.27 in women) (Table 3). The synergistic interaction of having diabetes and hypertension on stroke was only statistically significant in women (SI = 3.16, 95% CI: 1.35–7.39). The synergistic interaction of parental diabetes and being overweight on diabetes was only statistically significant in men, and not in women (SI = 3.30, 95% CI: 1.00–10.83 in men; SI = 1.15, 95% CI: 0.30–4.39 in women). Parental hypertension, in addition to being overweight, synergistically increased the likelihood of hypertension over that calculated for each factor alone (OR = 10.15, 95% CI: 6.20–17.85 in men; and OR = 10.08, 95% CI: 5.85–17.36 in women). The synergistic interaction of parental hypertension and being overweight on hypertension was statistically significant in both men and women (SI = 2.65, 95% CI: 1.18–5.96 in men; SI = 2.24, 95% CI: 1.05–4.79 in women).

**Table 3:** Multiple logistic regression and analyses for interaction effects of stroke, diabetes, and hypertension.

Outcome	Model	Gender	Risk factors		OR	(95%CI)	Synergic index	(95%CI)
			Diabetes	Hypertension				
Stroke	I	Male	-	-	1.00			
			-	+	3.20	(2.02–5.07)		
			+	-	2.17	(0.88–5.33)		
			+	+	3.91	(2.08–7.35)	0.86	(0.35–2.16)
	II	Female	-	-	1.00			
			-	+	6.03	(2.6–14.02)		
			+	-	1.16	(0.14–9.54)		
			+	+	17.42	(7.18–42.27)	3.16	(1.35–7.39)
			Parental diabetes	BMI≥24				
Diabetes	III	Male	-	-	1.00			
			-	+	2.48	(1.56–3.93)		
			+	-	5.78	(1.92–17.39)		
			+	+	21.64	(9.76–47.95)	3.30	(1.00–10.83)
	IV	Female	-	-	1.00			
			-	+	1.30	(0.80–2.11)		
			+	-	4.90	(1.86–12.89)		
			+	+	5.82	(2.60–13.05)	1.15	(0.30–4.39)
			Parental hypertension	BMI≥24				
Hypertension	V	Male	-	-	1.00			
			-	+	1.78	(1.34–2.37)		
			+	-	3.81	(2.06–7.06)		
			+	+	10.15	(6.20–17.85)	2.65	(1.18–5.96)
	VI	Female	-	-	1.00			
			-	+	2.01	(1.41–2.88)		
			+	-	4.04	(2.19–7.44)		
			+	+	10.08	(5.85–17.36)	2.24	(1.05–4.79)

BMI: body mass index. Model I included diabetes, hypertension, and age. Model II included diabetes, hypertension, and age. Model III included parental diabetes, BMI≥24, age, and hypertension. Model IV included parental diabetes, BMI≥24, age, and hypertension. Model V included parental hypertension, BMI≥24, age, and diabetes. Model VI included parental hypertension, BMI≥24, age, and diabetes.

## Discussion

Our study revealed gender differences in terms of the interaction effect of diabetes and hypertension on stroke in an elderly population. In both men and women, as with independent effects, hypertension is the strongest risk for stroke. A synergistic interaction for stroke was found when diabetes was combined with hypertension in elderly women. A synergistic interaction of parental diabetes and being overweight on diabetes were only found in men, and not in women. A positive parental history and being overweight had an additive effect on hypertension in both men and women. The findings of this study were strengthened by the large study population and comprehensive data analysis. The advantage of our study included using parental history as a proxy for genetic loading, synergy index analysis to determine the additive effect, and examining gender differences.

### Gender difference in terms of the synergistic interaction of HTN and DM on stroke

The gender difference of stroke are observed in the prevalence, incidence, and mortality of this condition [17–19]. Premenopausal women experience fewer strokes than age-matched men, but stroke rates increase among postmenopausal women compared with men of the same age. The mechanism that underlies the gender difference in stroke is complicated. Although some biological differences, such as sex hormones, body composition, and lifestyle may explain some differences [19], there is still much that is not understood. This could be due to estrogen deficiency. We had checked whether hormonal replacement will affect their outcome in females. However, the information of hormone therapy in our data is limited and the result is not significant. Peters et al. performed a meta-analysis of 64 cohorts and reported that the excess risk of stroke associated with diabetes is significantly higher in women than men, independent of sex differences in other major cardiovascular risk factors [20]. We found that diabetes and hypertension, major risk factors for stroke, had synergistic interaction on stroke in elderly women. Hu et al. investigated the interaction between diabetes and hypertension on the incidence of stroke and found that the two diseases increase the risk of stroke independently, but their combination greatly increases the risk [21]. No heterogeneity was found between sexes in that study as data on men and women were combined for the analysis. It has been reported that there is an additive interaction between hyperglycemia and hypertension in terms of their effects on the adhesion of endothelial cells, which may cause atherosclerosis and lead to further cardiovascular disease or stroke [17]. Taken together, our findings showed that risk factors have a different impact on cardiovascular risk in men compared to women. In women, arterial hypertension, diabetes, and their combination, has a greater risk in determining cardiovascular disease [22]. Our study confirmed a gender difference regarding the synergistic interaction of diabetes and hypertension on stroke, which explains part of the reason for the difference in the epidemiology of stroke between women and men. However, further work is needed to clarify the biological, behavior, or social mechanisms involved.

### Risks of diabetes

In the present study, men had a more pronounced synergistic interaction from the combination of parental history and being overweight than women. The risks of diabetes have been well investigated, and are likely to be mediated through a combination of genetic, anthropometric, and lifestyle factors, including BMI, dietary habits, physical activity, smoking, and alcohol consumption [5–7]. Diabetes risks will be improved if subjects monitor food intake and increase physical activity.

Previous studies that have investigated the interaction effect of parental history and being overweight on the risk of developing diabetes are scarce. Rising obesity rates are the greatest contributor to the increasing prevalence of type 2 diabetes in the United States, accounting for all of the increase in women and about half of the increase in men, as indicated by new comparative data from the National Health and Nutrition Examination Survey (NHANES) [23]. A possible underlying mechanism has been reported by previous studies that showed parental diabetes is associated with higher levels of insulin resistance, serum glucose, C-peptide, and leptin levels [24, 25]. Ning et al. studied 2,797 Chinese and 3,166 Finnish individuals aged 45–74 years, and found significant synergies between obesity (BMI ≥30 kg/m^2^) and a family history of diabetes in Finnish men, but no significant synergies were found in Chinese men [8]. Wikner et al. investigated 4,232 subjects aged 60-year-olds from Stockholm and found that obesity (BMI >30 kg/m^2^) in combination with the diagnosis of diabetes in one parent may be particularly hazardous in men as these two factors synergistically increase the risk of developing type 2 diabetes in men. No significant synergies were found between parental diabetes and being overweight (BMI >25 kg/m^2^) [9].

It has been reported that there are gender differences in terms of the risk factors for incident type 2 diabetes. Men and women have differing normal levels of blood pressure, smoking habits, alcohol consumption, and physical activity levels [26]. Furthermore, the levels of sex hormones also differ in men and women by different degrees at different ages. In addition to sex hormones, fat composition and insulin sensitivity were also different between sexes. Machann et al. assessed age- and gender-related differences in adipose tissue compartments and found that females are characterized by lower visceral and higher subcutaneous adipose tissue [27]. Therefore, men are more susceptible than women to the consequences of indolence and obesity, possibly due to differences in insulin sensitivity and regional fat deposition [28]. A study from Europe showed that men with parental diabetes are more likely to have impaired insulin sensitivity and beta cell function than women [29].

### Risks of hypertension

Few studies have investigated the interaction between parental hypertension and being overweight on hypertension. Wada et al. found that a family history of hypertension and obesity were detrimental factors that contributed to hypertension [10]. Simsolo et al. reported that a family history of hypertension and obesity are related to increased renal sodium reabsorption rates, which could explain the higher prevalence of hypertension in obese children [11]. Our findings also echoed these reports but further demonstrated a comparable synergistic interaction in both genders.

### Limitation

This study was conducted through home visits by well-trained interviewers and a reasonable correlation was present between the self-reported history and medical records [30]. Information about medication, laboratory data, and disease severity were not available. In addition, the intensity of smoking and alcohol consumption was not detailed and further analysis was therefore not available. Approximately 40% of the data regarding BMI was missing because the participants could not stand or sit on the weighing machine. Many of these were not overweight, which may have resulted in a bias towards the null and a consequent underestimate the magnitude of association. Parental history was measured using a self-reported questionnaire. The influence of recall bias must therefore be considered. One limitation is that patients with diabetes may have a better recall of a positive parental history than those without diabetes, which would cause an overestimation of the results.

### Clinical implications

According to our results, healthcare systems should pay closer attention to elderly women with diabetes and hypertension as they have additional risk for stroke. Glucose and blood pressure control is important to reduce their rate of cardiovascular complications. To prevent diabetes and hypertension in elderly individuals, we should pay closer attention to those who are overweight or obese and have a parental history because of the synergistic effects of being overweight and a positive parental history. It is important to keep an ideal body weight to avoid the high risks caused by synergistic interaction of being overweight and having a positive parental history. Our findings are important and practical as body weight is a modifiable risk in those with parental diabetes or hypertension.

## Conclusions

A positive parental history and being overweight were not only independently associated with an increased risk, but also had a synergistic interaction on diabetes or hypertension in an elderly population. Hypertension is the strongest risk factor for stroke, and synergistic interaction was found when combined with diabetes in elderly women. Strategies should be made to prevent individuals becoming overweight, especially in those with a positive parental history, in order to prevent not only diabetes and hypertension, but also further cardiovascular events, such as stroke.
